# Thresholds of Auditory-Motor Coupling Measured with a Simple Task in Musicians and Non-Musicians: Was the Sound Simultaneous to the Key Press?

**DOI:** 10.1371/journal.pone.0087176

**Published:** 2014-02-03

**Authors:** Floris T. van Vugt, Barbara Tillmann

**Affiliations:** 1 Lyon Neuroscience Research Center, Auditory Cognition and Psychoacoustics Team, CNRS-UMR 5292, INSERM U1028, University Claude Bernard Lyon-1, Lyon, France; 2 Institute of Music Physiology and Musicians' Medicine, University of Music, Drama and Media, Hannover, Germany; Northwestern University, United States of America

## Abstract

The human brain is able to predict the sensory effects of its actions. But how precise are these predictions? The present research proposes a tool to measure thresholds between a simple action (keystroke) and a resulting sound. On each trial, participants were required to press a key. Upon each keystroke, a woodblock sound was presented. In some trials, the sound came immediately with the downward keystroke; at other times, it was delayed by a varying amount of time. Participants were asked to verbally report whether the sound came immediately or was delayed. Participants' delay detection thresholds (in msec) were measured with a staircase-like procedure. We hypothesised that musicians would have a lower threshold than non-musicians. Comparing pianists and brass players, we furthermore hypothesised that, as a result of a sharper attack of the timbre of their instrument, pianists might have lower thresholds than brass players. Our results show that non-musicians exhibited higher thresholds for delay detection (180±104 ms) than the two groups of musicians (102±65 ms), but there were no differences between pianists and brass players. The variance in delay detection thresholds could be explained by variance in sensorimotor synchronisation capacities as well as variance in a purely auditory temporal irregularity detection measure. This suggests that the brain's capacity to generate temporal predictions of sensory consequences can be decomposed into general temporal prediction capacities together with auditory-motor coupling. These findings indicate that the brain has a relatively large window of integration within which an action and its resulting effect are judged as simultaneous. Furthermore, musical expertise may narrow this window down, potentially due to a more refined temporal prediction. This novel paradigm provides a simple test to estimate the temporal precision of auditory-motor action-effect coupling, and the paradigm can readily be incorporated in studies investigating both healthy and patient populations.

## Introduction

Many motor actions have sensory consequences. For example, we see our hands displace when we move them, and our steps make sounds. The human brain is able to predict the sensory effects of its actions [1]–[3]. These predictions are crucial for distinguishing between sensory information that is generated by oneself and sensory information coming from outside. In particular, self-produced sensory effects are suppressed in comparison with externally produced effects [4].

The brain is able to predict not only *what* sensory event will follow its action, but also *when* it is supposed to occur. This is evident from the observation that self-produced sensory effects are no longer suppressed when they are delayed by several hundreds of milliseconds [5]. Furthermore, the temporal prediction is not fixed, but adaptive to the situation. For example, the point of subjective synchrony (PSS) between various sensory events can be recalibrated, even to the extent that the physical order of events can be inverted [6]–[9]. Lesions may affect subjective synchrony as shown by the intriguing case of a man who hears people speak before their lips move [10]. Synchrony can also be recalibrated between sensory and (active) motor events [11]–[14].

But how precise are these predictions and the perceived synchrony between pairs of sensory events, or motor and sensory events? Common experimental paradigms to measure this precision are asking participants to either judge whether two stimuli are simultaneous (simultaneity judgement task - SJ) or to report the order of two stimuli (temporal order judgement - TOJ). For the temporal order judgement task (TOJ), precision is measured as the just-noticeable difference (JND) between two potential orderings. Asynchrony detection thresholds vary according to the sensory modalities that are tested. For instance, humans can distinguish two auditory clicks presented to the same ear when they are separated by 2 msec, but at least 60 msec are needed to distinguish them binaurally [15]. Typical thresholds for TOJ between two auditory stimuli are inter-stimulus-intervals (ISIs) of 20 to 60 msec, probably depending on the stimulus type [16]–[18]. Thresholds for TOJ between auditory (tone) and visual (flash) stimuli are typically between 25 and 50 msec [19], [20]. Auditory-haptic thresholds usually have JNDs of 100 msec, and haptic-haptic thresholds have JNDs of around 50 msec [21]. Although the SJ and TOJ tasks often give different results, thresholds for the SJ task tend to be smaller than those for the TOJ task [22], [23]. This led to the dominant view that the SJ and TOJ tasks probably measure different underlying processes [24]. Furthermore, training plays a role in shaping sensitivities, as is shown by video game players having smaller thresholds for audio-visual simultaneity judgements than non-players [25].

It remains unclear how sensitive participants are to the synchrony between events that they actively produce (such as keystrokes) and their sensory consequences (such as tones). Previously, this question has been studied by investigating the effects of altered sensory feedback to a produced action. For instance, musicians' timing performance was measured when they played on a piano that emitted the played sounds with a delay. Large delays (such as 200 msec) are noticeable and disrupt the fluidity of performance [26]–[28]. Speakers' fluency is similarly affected when auditory feedback is delayed [29]–[32]. In order to be able to assess quantitatively whether disruptions in auditory feedback are noticeable and to investigate the effect of training and expertise, there is a need for an experimental paradigm that can establish thresholds for action-effect synchrony judgements.

The present research proposes a new tool to measure thresholds between a simple action and an emitted sound. In this task, participants are asked on each trial to press a key. Either immediately or after a predetermined duration has elapsed, a sound is presented through participants' headphones. Our aim was to measure the thresholds for detecting a delay between the keystroke and the sound, and to investigate the effect of expertise. In addition, our aim was to establish how this action-effect synchrony sensitivity relates to other auditory and auditory-motor capacities. To this end, our participants also performed, firstly, an auditory temporal deviant detection task, and secondly, a sensorimotor synchronisation task. That is, we measured how well they could synchronise their movements to an external stimulus [33], [34]. For this, we used a variation of the synchronisation-continuation tapping paradigm [35], [36]. All tasks were performed by non-musicians and by pianist and brass player musicians. It has been previously reported that musicians outperform non-musicians in terms of improved auditory discrimination [37], [38], and by tapping closer to the beat and more precisely [39], [40]. We further hypothesised that the relation between finger movements and sounds for the musicians' main instrument might influence the delay detection thresholds too: when pianists strike a key the sound is instantaneous, whereas brass players' sound onset is determined by their respiration. Also, the piano sound has a sharper onset than the brass sound. As a result, we expected that pianists would have lower thresholds than brass players. We had also considered singers as an alternative to the brass players, but found that they tend to have a large amount of piano training as their secondary instrument, which would be a confound for the comparison with pianists.

## Materials and Methods

### Ethics statement

The experiment was approved by the ethics committee of the University of Music, Drama and Media and were in line with the declaration of Helsinki. Participants provided informed written consent.

### Participants

We recruited two groups of musician participants from the student pool at the Hanover Music University and young professionals. We furthermore recruited non-musicians in the same age range. Table 1 lists biographical and questionnaire data of each group. Participants reported no hearing impairment or neurological disorder, were aged between 18 and 40 years and right-handed. The musician participants were recruited in two groups: one group whose primary instrument was the piano (or who were professional pianists) and another group with a main instrument from the brass family (e.g., trumpet, trombone or tuba). A further criterion for inclusion in the non-musician group was having received less than 1 year of musical training (apart from obligatory courses in primary or secondary schools).

**Table 1:** Basic information about the three groups of participants.

	Pianists	Brass	Nonmusicians	
N	20	18	18	
Gender (female/male)	10/10	7/11	8/10	χ^2^(2) = .47, p = .79
Age (years)	26.1 (5.7)	24.9 (3.5)	26.2 (4.7)	Kruskal-Wallis χ^2^(2) = 1.07, p = .59
Handedness (Handedness Quotient in %)	73.4 (19.9)	75.3 (16.5)	78.1 (20.0)	Kruskal-Wallis χ^2^(2) = 1.10, p = .58
Capable of blind typing (number of participants in each of the following categories: 10 fingers/less than 10 fingers/none)	2/14/4	7/10/1	5/10/3	Kruskal-Wallis χ^2^(2) = 4.67, p = .10
Video game use in hours per week (number of participants in each of the following categories: none/<1 h/1–7 h/>7 h)	16/3/1/0	10/7/1/0	13/1/3/1	Kruskal-Wallis χ^2^(2) = 2.09, p = .35
Use of computer keyboards in hours per day (number of participants in each of the following categories: <1 h/1–2 h/>2 h)	10/8/2	7/9/2	5/2/11	Kruskal-Wallis χ^2^(2) = 7.84, p = .019*
Capacity in using computer keyboards (self-rated 1–10)	6.3 (1.6)	6.6 (1.9)	6.7 (2.1)	Kruskal-Wallis χ^2^(2) = 0.87, p = .65
Use of text messaging on cell phone in hours per week (number of participants in each of the following categories: none/<1 h/1–7 h/>7 h)	7/9/4	4/12/2	10/4/4	Kruskal-Wallis χ^2^(2) = 1.42, p = .49
Capacity in using text messaging (self-rated 1–10)	7.3 (2.0)	6.8 (1.9)	6.2 (2.3)	Kruskal-Wallis χ^2^(2) = 2.41, p = .30
Age of onset of musical training (years)	6.65 (2.2)	9.78 (3.1)	NA	t(30.5) = −3.56, p = .001**
Accumulated practice time on principal instrument (×10,000 hours)	22.6 (10.5)	13.1 (8.1)	NA	t(35.3) = 3.15, p = .003**
Years of musical practice	19.5 (5.6)	15.1 (3.6)	NA	t(32.6) = 2.90, p = .007**
Current daily practice time (hours)	3.7 (2.2)	3.3 (1.8)	NA	t(35.6) = 0.68 p = .50
Absolute hearing (yes/no; self-reported)	7/13	0/19	NA	Fisher Exact Test p = .009**

Data is reported as mean (SD) unless otherwise specified. Uncorrected significance is indicated: *p<.05, **p<.01, ***p<.001.

Among the brass players, 13 had received piano instruction in the form of obligatory courses at the conservatory or in their childhood. For the entire brass group, the lifetime accumulated piano practice time was 1.1 (SD 1.8) thousand hours over an average total of 6.9 (SD 6.1) years.

Participants filled out a questionnaire with basic information such as age, handedness (according to the Edinburgh Handedness Inventory), and instrumental practice prior to their participation. Questionnaire results are reported in Table 1. We found that basic biographical parameters did not differ except for computer keyboard use (Kruskal-Wallis χ^2^(2) = 7.84, p = .019 uncorr.), This effect indicated that the non-musician group reported they spent more time in a day using a computer keyboard than the pianists [Mann-Whitney U = 98.0, Z = 2.55, p = 0.01, r = 0.07] or brass players [Mann-Whitney U = 96.5, Z = 2.20, p = 0.03, r = 0.06]. The two musician groups did not differ in their use of computer keyboards [Mann-Whitney U = 161.0, Z = 0.61, p = 0.54, r = 0.02].

### Materials

#### Keystroke-sound delay detection task

We used a USB keypad (Hama Slimline Keypad SK110) that interfaced through the HDI protocol with a python script. This script detected keystroke onsets and played a woodblock wave sound (duration: 63 msec) after a predetermined duration through headphones (Shure SRH440). The woodblock sound was chosen because of its relatively sharp sound onset and nevertheless being pleasant to hear.

#### Anisochrony detection

We used a python-pygame graphical user interface that presented the sounds (using pyAudio) and the instructions. Instructions were given orally as well. The five-tone sequences were generated as follows (adapted from [37], [41]). The base sequence consisted of five isochronous sine wave tones of 100 ms presented with an inter-onset-interval (IOI) of 350 ms. In some trials, the fourth tone was delayed by a certain amount but the fifth tone was always on time [37], [41]. That is, when the tone was delayed by an amount *d*, the third interval was longer by *d* msec and the fourth interval was shorter by *d* msec.

#### Synchronisation-Continuation Tapping

The synchronisation stimulus was generated offline as follows and saved to a wave file. First, we presented 4 finger snap sounds with an inter-onset-interval of 300 msec. Then 30 instances of the woodblock sound (the same as used during the learning part of the experiment) followed with an inter-onset-interval (IOI) of 600 msec. This was followed by a silence of 30*600 msec (the equivalent of 30 more taps). Finally, a high-pitched gong sound was used to signal the end of the trial. The sounds were played using a custom developed python experimental script, which also communicated through a HID-USB interface with the button box to register the responses.

Participants' finger taps were recorded using a custom tapping surface containing a (piezo-based) contact sensor that communicated with the computer through the serial interface and was captured in a python program that also presented the stimuli using pyAudio.

### Procedure

#### Keystroke-sound delay detection task

In the delay detection task, we measured participants' sensitivity to delays between motor (keystroke) and auditory (tone) events. That is, we established from which delay onwards participants noticed that the tone came after the keystroke instead of immediately. At each trial, the participant pressed the “zero” key on the keypad at a time of her/his choosing and heard a tone. This tone was either played at the same time of the keystroke or temporally delayed. The participants responded verbally whether or not they had the feeling that the tone was delayed. Their responses were entered in the computer by the experimenter. Crucially, participants were instructed to leave their finger on the key (instead of lifting it prior to the keystroke) so as to reduce the tactile timing information. Furthermore, they were required to keep their eyes closed during the keystroke.

We used the Maximum Likelihood Procedure (MLP) algorithm [42]–[45] to establish the threshold for the detection of the asynchrony between movement (keypress) and the tone. The algorithm is designed to adaptively select the stimulus level (tone delay) on each trial so as to converge to the participants' threshold. For each block, the algorithm outputs an estimate for the participant's threshold.

The MLP algorithm briefly works as follows. Participants' probability of responding “delayed” to a particular stimulus (i.e. keystroke-sound delay) is modeled by sigmoid psychometric curves that take stimulus level (amount of delay in msec) as a variable. The equation for the psychometric curves was p(response delayed) = a+(1−a)*(1/(1+exp(−k*(x−m)))), where a is the false alarm rate (see below), k is a parameter controlling the slope, m is the midpoint of the psychometric curve (in msec) and x is the amount of delay (in msec). A set of candidate psychometric curves is maintained in parallel and for each curve, the likelihood of the set of the participants' responses is calculated. The psychometric curve that makes the participant's responses maximally likely is used to determine the stimulus level (the delay between the keystroke and the sound) on the next trial. We used 600 candidate psychometric curves with midpoints linearly spread between 0 and 600 ms delay and combined these with the five false alarm rates (0%,10%,20%,30%,40%). Hence, a total of 3000 candidate psychometric curves were used.

Participants first performed 4 trials (2 with no delay and 2 with a delay of 600 ms) to make clear the difference between when the sound came immediately and when it was delayed. The participant received accuracy feedback about her answers during these practice trials. Next, they performed a block of 10 trials, starting at a 600 ms keystroke-sound delay but then using MLP to determine the stimulus levels of the following trials. If the procedure was clear, we continued with 3 experimental blocks of 36 trials. Each experimental block consisted of 36 trials containing 6 catch trials. Catch trials are trials on which the delay was always 0 msec (regardless of the delay that was suggested by the MLP algorithm). The function of catch trials is to prevent participants from always responding “delayed” (which would cause the MLP algorithm converge to a zero threshold). Catch trials were inserted randomly with the following constraints: the first 12 trials contained 2 catch trials and the next 24 trials contained 4 catch trials.

The maximum likelihood procedure was implemented in python. We made our source code freely available online on https://github.com/florisvanvugt/PythonMLP. The source code for the delay detection paradigm is furthermore available upon request (to the corresponding author).

#### Anisochrony detection

Participants were seated comfortably and on each trial heard a sequence of five tones (see materials). Participants' task was to respond whether the five-tone sequence was regular or not by pressing one of two response keys on the laptop keyboard. Stimuli (see materials) were presented through headphones set to a comfortable sound level that was kept constant across all participants. The participant's threshold was established adaptively using the MLP procedure. The basic procedure was the same as for the delay detection task, but here the set of candidate psychometric curves was as follows. We defined 200 logistic psychophysical curves whose midpoints were linearly spread over the 0 to 200 ms delay range (0% to 57% of the tone IOI) and these were crossed with the five false alarm rates (0,10,20,30,40%). Again, each experimental block consisted of, first, 12 trials containing 2 catch trials, and then 24 trials containing 4 catch trials.

Instructions were presented orally and then written on the screen. Next, the interface presented the four example stimuli (two regular, two irregular). For these trials, participants received accuracy feedback. The first trial of the next block of 10 trials was set to a keystroke-sound 200 msec delay and then the adaptive procedure (MLP) was used to determine the stimulus level on the next trials. During this second training block, no accuracy feedback was provided. Finally, if the procedure was understood by the participants, three experimental blocks were administered. In between blocks, participants took a brief break of several minutes.

#### Synchronisation-Continuation Tapping

In each trial, participants tapped with their index finger on a flat surface along with the synchronisation stimulus after the four finger snap sounds (see materials). When the woodblock sounds stopped, participants were instructed to continue tapping at the same speed and regularity until the high-pitched sound signalled the end of the trial.

#### Data analyses

The threshold tasks were analysed as follows. First, we discarded blocks that contained more than 30% incorrect catch trial responses (in which the delay or deviation was 0 msec). Secondly, we discarded blocks in which the threshold estimate had not properly converged towards the end of the block. This was tested by fitting a regression line to the last 10 trials in the block, and discarding those blocks in which the slope of this line exceeded 2 msec/trial (for the delay detection task) or 1.18 msec/trial (for the anisochrony task). These slope cut-off points were chosen so as to, firstly, match visual inspection of blocks that had not properly converged, and secondly, to be roughly the same proportion of the average final threshold in the anisochrony and delay detection task. Thirdly, we computed the average threshold estimate for the remaining blocks for each participant.

Synchronisation tapping performance was analysed using linear and circular statistics. In the linear analysis, we calculated the time between each tap and its corresponding metronome click (in msec). For each block, we averaged these to yield the mean relative asynchrony (in msec) and calculated the standard deviation (SD) to yield the SD of the relative asynchrony (in msec). The mean relative asynchrony is a measure for how close participants tapped to the beat and the SD relative asynchrony is a measure of tapping precision (time-lock). In the circular analysis [46], the timing of each tap was converted into a phase (between 0 and 2π) relative to the metronome onset. Based on these, we calculated the synchronisation vector, which is the average of all vectors with length 1 and the phase angle for that tap. The length of this vector (between 0 and 1) is a measure for the time-lock between the tap and the sound. We used Fisher's r-to-z transformation and fed the obtained z-scores into our parametric analysis.

For the continuation phase (when the metronome had stopped), we calculated the intervals between taps (inter-tap-interval, ITI, in ms) and its standard deviation (SD ITI in ms). We then de-trended the continuation taps by fitting a regression line to the ITIs over time, reporting the slope of this line and taking the residual variability from this line. In this way, we compensated for the fact that participants tend to speed up or slow down [47]–[49]. The slope of this line fit indicated the tempo drift.

In order to compare performance of the three groups, we performed between-participants ANOVAs. We tested for homogeneity of variance using Levene's Test, and report where it was significant. We report generalised effect sizes η_G_^2^ [50]. Follow-up comparisons were calculated using Tukey's HSD method.

The data collected within the framework of this study are made available freely online (http://dx.doi.org/10.6084/m9.figshare.878062).

## Results

### Delay Detection

We discarded 17.0% of all blocks because of catch trial errors, and a further 2.3% because of lack of threshold convergence. Four participants (2 pianists, 2 brass players) had no remaining blocks and were eliminated from further analyses. For the other participants, we calculated the average of the thresholds on the basis of the remaining 2.6 (SD 0.7) blocks.

**Figure 1:**
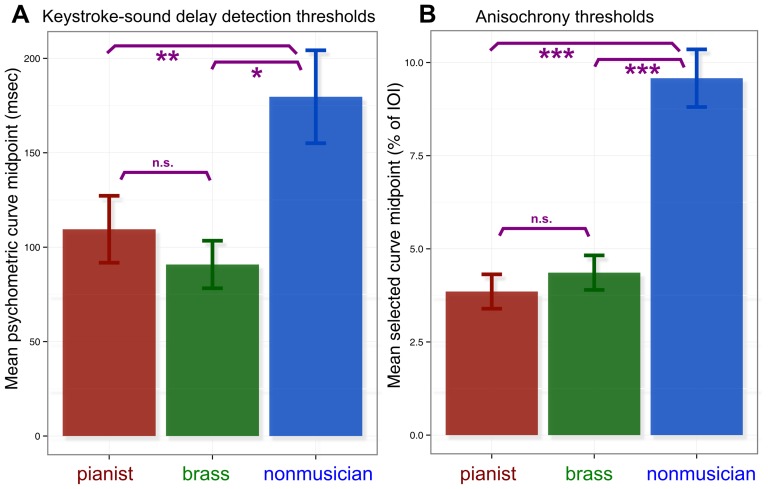
Thresholds for the keystroke-sound delay detection (A) and anisochrony (B) tasks. The figures indicate the average thresholds for each of the groups (error bars indicate the standard error of the mean). *p<.05, **p<.01, ***p<.001.

The distribution of thresholds of all participants in all groups combined was significantly non-normal [Shapiro-Wilk normality test W = .86, p = .00003], and therefore we continued statistical analyses with log-transformed thresholds. These did not violate normality assumptions [Shapiro-Wilk W = .98, p = .71]. The main effect of group (pianist, brass, nonmusician) on delay detection threshold was significant [F(2,49) = 6.40, p = .003, η_G_^2^ = .21]. Post-hoc Tukey HSD contrasts indicated that the non-musicians' threshold was higher than those of the pianists [p = .01] and than those of the brass players [p = .006]. The brass players and pianists' thresholds were not significantly different [p = .93] (Figure 1A). Among the brass players, we found that those who played piano as their second instrument (N = 11) had a lower delay detection threshold (M = 83.0, SD = 42.5) than those who did not (N = 5) (M = 116.2, SD = 70.0). However, this difference was not significant [t(7.3) = 1.09, p = .31]. Furthermore, the brass players that did not have piano as their second instrument (N = 5) did not show a higher delay detection threshold than pianists [t(7.8) = −.50, p = .63].

### Anisochrony

We discarded 11.1% of all blocks because of catch trial errors, but no further blocks were discarded because all had properly converged. Two participants (1 brass, 1 pianist) had no blocks remaining (based on the first criterion) and were eliminated from further analyses. For the other participants, we averaged the remaining 2.7 (SD = 0.6) blocks into a single threshold value per participant.

The distribution of thresholds was significantly non-normal [Shapiro-Wilk normality test W = .92, p = .0009] and therefore we continued statistical analyses with log-transformed thresholds. These did not violate normality assumptions [Shapiro-Wilk W = .97, p = .20]. The main effect of group on anisochrony threshold was significant [F(2,51) = 21.60, p<.0001, η_G_^2^ = .46]. Tukey HSD contrasts indicated that nonmusicians' thresholds were higher than those of the pianists [p<.001] and than those of the brass players [p<.0001]. The brass players' and pianists' thresholds did not differ significantly [p = .52] (Figure 1B). In the pianist group, there was one outlier who was further than 3 SD below the mean for that group, but removing this participant did not affect any of the results.

### Synchronisation-Continuation Tapping

We report basic measures of synchronisation and tapping variability in Table 2. Tukey contrasts revealed that brass players and pianists do not differ in any of the measures (all p>.73) but contrasts between the non-musicians on the one hand and the pianist or brass groups on the other yielded significant or marginally significant differences (all p<.08) (Table 2).

**Table 2:** Synchronisation and continuation tapping results for the three groups.

	Pianists	Brass players	Nonmusicians	Between-groups comparison
*Synchronisation phase*				
Mean relative asynchrony (msec)	7.5 (21.2)	5.7 (28.3)	−22.5 (52.3)	F(2,44) = 3.35, p = .04*
SD relative asynchrony (msec)	19.9 (4.9)	19.3 (3.2)	37.4 (20.3)	F(2,44) = 11.7, p<.0001
Synchronisation vector length (r-bar, z-transformed)	2.3 (0.2)	2.3 (0.2)	1.8 (0.4)	F(2,44) = 20.85, p<.00001
*Continuation phase*				
Continuation ITI (msec) (without detrending)	604 (10)	605 (11)	596 (20)	F(2,44) = 1.79, p = .18+
Continuation SD ITI (msec) (without detrending)	17.9 (2.9)	19.6 (2.7)	31.4 (9.0)	F(2,44) = 27.04, p<.000001
Continuation drift (msec/sec)	−0.3 (0.6)	−0.4 (0.8)	−0.9 (1.0)	F(2,44) = 2.65, p = .08.
Continuation residual variability after detrending (CV %)	5.5 (0.9)	6.0 (0.8)	9.7 (2.9)	F(2,44) = 25.7, p<.00001, etasq = .54

Values are reported as mean (SD) unless otherwise specified.

+ For the continuation ITI, Levene's test for homogeneity is violated.

### Comparisons between the tests

Participants' performances on the various tests reported here were not independent. Combining the thresholds from the three groups, the delay detection threshold correlated positively with the anisochrony task [Pearson ρ(49) = .60, p<.0001, R_adj_^2^ = .35]. The delay detection threshold correlated negatively with the synchronisation vector length [Pearson ρ(49) = −.53, p<.0001, R_adj_^2^ = .27].

To test whether these correlations differed statistically between the groups, and whether performance on the anisochrony and synchronisation tasks combined might explain more of the variance in delay detection than either of those two tasks alone, we performed the following analysis. Participants who had at least one valid anisochrony block and at least one valid delay detection block remaining (after discarding) entered in this analysis. This was the case for 17 pianists, 16 brass players and 18 non-musicians. We ran an ANCOVA model with log-transformed delay detection threshold as dependent variable, group (nonmusician, brass player or pianist) as categorical factor (between-participants) and log-transformed anisochrony threshold and sensorimotor synchronisation accuracy (vector length, r-bar) as covariates.

**Figure 2:**
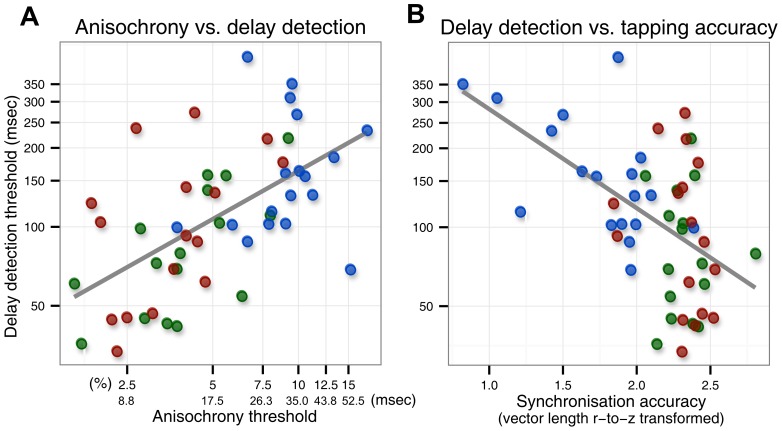
Correlations between keystroke-sound delay detection and anisochrony (A) and sensorimotor synchronisation accuracy (B). The dot colour indicates the group: blue for non-musicians, red for pianists and green for brass players.

The interaction between anisochrony threshold and group was not significant [F(2,48) = 1.64, p = .21], which indicated that the linear relationship between the anisochrony and delay detection thresholds were not different between the groups. The interaction between synchronisation accuracy and group was not significant either [F(2,48) = .91, p = .41]. This means that the linear relationship between synchronisation accuracy and delay detection was not different between groups. The main effect of anisochrony threshold was significant [F(1,48) = 5.56, p = .02] as was the main effect of synchronisation accuracy [F(1,48) = 8.73, p = .004]. There was no main effect of group [F(2,48) = 1.06, p = .35]. These results were essentially the same when repeated without the participant with an outlier anisochrony threshold (Figure 2).

In sum, the anisochrony and synchronisation accuracy both significantly explained the variance in delay detection thresholds (Figure 2). Taken together, they explained more than either one factor alone. With these two predictors, the group (pianist, brass, nonmusician) factor did not explain additional variance, indicating that the musicianship effect on delay detection threshold was explained by anisochrony and synchronisation task performance.

## Discussion

The human brain predicts sensory effects of its motor actions [1], [51]. Not only does the brain predict *what* effect will follow, but also *when* it is expected to occur [5]. The present paper presents a simple test to measure the precision of this temporal prediction window. We applied this test to a non-musician population and two groups of musicians: brass players and pianists in order to investigate the effect of training. We furthermore asked how the sensitivity to auditory-motor delays builds on other auditory and auditory-motor tasks.

Our findings suggest that the brain has a relatively large window of integration (102±65 ms for musicians, and 180±104 ms for nonmusicians) within which an action and its resulting effect are judged as simultaneous. These delay detection thresholds are larger by almost an order of magnitude than thresholds for judging two auditory events as asynchronous, which are between 2 and 60 msec [15]–[18]. However, the present findings are in line with cross-modal sensory asynchrony judgements: visual and auditory events simultaneity thresholds are usually around 150 ms [52].

Participants' capacity to judge simultaneity of movement and sound can be explained as a combination of auditory temporal prediction precision (anisochrony) and sensorimotor synchronisation accuracy. That is, the delay detection task appears to tap into basic cognitive capacities of auditory processing and auditory-motor coupling. Both of these capacities varied with musicianship, and the latter did not additionally explain variance in the thresholds of audio-motor synchrony judgements.

These results suggest that, first of all, sensitivity to auditory-motor delays can be trained. Musicians were more precise in temporally predicting the auditory effect of their movement, as evidenced by their lower threshold in the delay detection task. This finding is in line with the finding that musical training improves performance in a variety of tasks [37]–[40], [53] and also induces functional and structural brain changes [54], [55]. In addition, the finding is in line with previous studies showing that temporal order judgements (TOJ) improve with training [56], [57]. However, a limitation to our present study is that we cannot conclude whether musicianship caused lower delay detection thresholds, or vice versa. It is conceivable that people with lower delay detection thresholds enrolled in musical training more than those who had higher delay detection thresholds. In order to conclusively answer this question, a future longitudinal study could follow a sample of participants and randomly assign them to music- or other (control) training. If such a study would find a reduction in delay detection threshold in the group participating in musical training, but not in the control group, this could prove that delay detection thresholds are lowered as a result of musical training.

Secondly, musicianship appears to improve delay detection thresholds indirectly. That is, musicianship did not significantly influence delay detection sensitivity when performance on purely auditory (anisochrony) or auditory-motor tasks (sensorimotor synchronisation) was taken into account. This means that auditory-motor delay detection is not a capacity that is specifically improved by music training. If this were so, we would have expected to find differences in correlations between the tests (delay detection, anisochrony and sensorimotor synchronisation) between our groups. This was not the case. Instead, the results suggest that musical training improves sensorimotor synchronisation capacities as well as auditory temporal precision, both of which then lead to an improvement in delay detection threshold. A potential alternative explanation for our finding is that musicianship affects a latent variable (or latent variables), not measured here, and that this variable improves delay detection sensitivity, auditory temporal precision and sensorimotor synchronisation.

Furthermore, the instrument that musicians played had no influence on delay detection sensitivity, or any of our other tasks. This suggests that the specifics of how an instrument responds to finger movements of the musician nor the acoustic features of the instrumental sound influence the capacity to detect delays between movement and sound.

Humans' conscious sensitivity to delays between their articulator movements and the produced speech sound is typically around 60–70 msec [14], but implicit adjustments of speech rate to delayed feedback are reported from 50 msec delay onwards [32]. These delays are below the thresholds observed here, but close to the thresholds we found for musicians. Humans accumulate many hours of speech practice (many more than even professional musicians could accumulate on their instrument) and therefore one will expect to find lower delay detection thresholds for vocal actions. This finding squares with the idea that training an action, be it speaking or playing an instrument, improves the temporal prediction of its sensory consequences. However, the particular instrument that the musicians trained to play (piano or brass instruments) did not influence sensitivity, suggesting that perhaps delay sensitivity is specific to the effector: the articulators in the case of speech and the hand in the case of piano playing and brass playing, and perhaps also the mouth in the case of brass playing. Notice, however, that comparisons between music and speech are limited by the fact that there exist no control group with negligeable speech experience.

The present study has some limitations. It might be argued that the experimental setup of this study involves an inherent delay between the keystroke and the sound. Possibly, musicians who were exquisitely sensitive to delays considered even the shortest possible latency in our setup as asynchronous. However, if this were the case we would have expected participants to exhibit thresholds close to zero, which was not the case. Furthermore, as we have argued above, the thresholds we found for musicians were comparable to those found in speech.

A limitation of our comparison between pianists and brass players is that the difference between those groups might have been reduced due to the fact that many brass players had some piano experience. This is not a bias in our sample, but reflects the reality of musical education in which musicians are encouraged to practice a secondary instrument, and piano is a popular choice. Crucially, we found no differences in a post-hoc comparison among brass players between those with piano experience and those without it. Furthermore, the brass players without piano experience did not differ from the pianists.

Future studies could use the delay detection task to tap into temporal prediction capacities to investigate auditory-motor processing. The paradigm could also provide a precise quantification of temporal binding, which is the phenomenon that a person's self-generated sensory stimuli appear closer in time to the action that caused them than externally-generated sensory stimuli [58].

## Conclusions

The present findings suggest that the brain has a relatively large window of integration within which an action and its resulting effect are judged as simultaneous. Furthermore, musical expertise may narrow this window down, potentially due to more refined general temporal prediction capacities and improved auditory-motor synchronisation (as suggested by the data of anisochrony and sensorimotor synchronisation tasks, respectively). The presently proposed paradigm provides a simple test to estimate the precision of this prediction. Musicians' temporal predictions were more precise than that of nonmusicians, but there were no reliable differences between pianists and brass players. The thresholds correlated with a purely auditory threshold measure requiring the detection of a temporal irregularity in an otherwise isochronous sound sequence. Furthermore, they correlated with sensorimotor synchronisation performance. This suggests that musical training improves a set of auditory and auditory-motor capacities. These capacities are then used together to generate temporal predictions about the sensory consequences of our actions. The particular instrument as well as practice time has only a minor influence. This novel paradigm provides a simple test to estimate the strength of auditory-motor action-effect coupling that can readily be incorporated in a variety of studies investigating both healthy and patient populations.
